# Prevalence of malnutrition among Samoan children aged 5 to 11 years in 2019–2020

**DOI:** 10.1080/03014460.2023.2197298

**Published:** 2023-02

**Authors:** Courtney C. Choy, Vaimoana Filipo Lupematasila, Maria Siulepa Arorae, Faatali Tafunaina, Folla Unasa, Christina Soti-Ulberg, Muagututia S. Reupena, Rachel L. Duckham, Kima Faasalele-Savusa, Take Naseri, Nicola L. Hawley

**Affiliations:** a Department of Chronic Disease Epidemiology, yale school of Public Health, new Haven, CT, USA;; b Obesity, Lifestyle, and Genetic Adaptations Study Group, Apia, Samoa;; c Ministry of Health, Apia, samoa;; d Lutia i Puava ae mapu I fagalele, Apia, samoa;; e Institute for Physical Activity and nutrition (IPAN), Deakin university, Burwood, Australia;; f Australian institute for musculoskeletal science, university of melbourne and Western Health, st Albans, Australia;; g Department of Epidemiology, school of Public Health, Brown university, Providence, RI, USA

**Keywords:** Obesity, stunting, anaemia, children, Samoa

## Abstract

**Background:**

Globally, rapid economic development, urbanisation, and nutrition transitions have led to rising levels of malnutrition in all forms.

**Aim:**

The study objective was to document the prevalence of overweight/obesity, underweight, stunting, and anaemia among Samoan children in 2019–2020.

**Subjects and methods:**

Children from the *Ola Tuputupua’e* “growing up” in Samoa study at ages 5–11 years with complete physical assessments were included. overweight/obesity, underweight, and stunting were classified using World Health organisation Z-scores for body mass index-for-age (BMIZ> +1), weight-for-age (WAZ< –2SD), and height-for-age (HAZ< –2SD), respectively. Anaemia was defined as haemoglobin concentration <11.5 g/dl. Prevalence was compared by child age, sex, and census region of residence (representing urbanicity and exposure to nutrition transition) using Wilcoxon two-sample, chi-square, or Fisher’s exact tests.

**Results:**

The prevalence of overweight/obesity, underweight, stunting, and anaemia was 36.2%, 0.5%, 1.6%, and 31.6%, respectively. overweight/obesity in children was positively associated with age and highly prevalent in periurban and urban regions. While children living in the rural region with the lowest exposure to nutrition transition had the highest prevalence of mild-to-moderate stunting, anaemia prevalence was lower compared to those in the urban region. no sex differences in malnutrition were observed.

**Conclusion:**

Moderate-to-high levels of overweight/obesity and anaemia call for comprehensive intervention strategies.

## Background

Young children with malnutrition begin their lives with marked disadvantages: they face developmental delays and experience adverse health outcomes that may lead to learning difficulties in school and lesser earning potential as adults (UNICEF, WHo, & World Bank 2021). at one end of the malnutrition spectrum, overweight/obesity during childhood can increase the risk of obesity and premature development of non-communicable, cardiometabolic diseases in adolescence and adulthood (Dietz 1998). on the other, undernutrition, both in protein-energy and micronutrients, increases the severity of illness and symptoms of infection, like diarrhoea (Black et al. 2008). children are particularly vulnerable to the double burden of overweight/obesity and undernutrition because of nutritional transitions (Black et al. 2017; Britto et al. 2017). coinciding with rapid economic development and epidemiological transitions towards an increasing prevalence of non-communicable diseases, nutrition transition involves shifts in dietary patterns towards increasing energy-dense, nutrient-poor foods, alongside more sedentary lifestyles (Doak et al. 2005; Wells et al. 2020).

Studies conducted in Samoa over the past three decades have documented significant economic and nutrition transitions (galanis et al. 1999; Seiden et al. 2012) and an increasing burden of malnutrition starting in early childhood (choy et al. 2017; choy et al. 2020). among Samoan children aged 2–4 years old in 2015, moderate levels of overweight/obesity (Bmi-for-age z-score > +2 based on World Health organisation (WHo) growth standards; 16.1%), stunting (height-for-age z-score < −2; 20.3%) and anaemia (Haemoglobin <110 g/dl; 34.1%) were reported (choy et al. 2017). While increases in overweight/obesity and a persistent, moderate level of anaemia were observed among these Samoan children at age 4–6 years, there has been no strong evidence to support a relationship between overweight/obesity and anaemia in early childhood (choy et al. 2022). The purpose of this study was to provide an update in an expanded cohort of these same Samoan children and document the prevalence of malnutrition (overweight/obesity, underweight, stunting, and anaemia) at ages 5–11 years old in 2019–2020.

### Sample

Data were obtained from the *Ola Tuputupua’e* “growing up” in Samoa project, a mixed longitudinal cohort study to understand childhood growth, development, and health. initial recruitment of the cohort has been described (choy et al. 2017). Briefly, a convenience sample of 319 child-primary caregiver dyads was recruited from 10 villages in the island of ‘Upolu in 2015. villages were selected to represent three census regions and differential exposure to the nutrition transition based on urbanicity: the rest of Upolu (*n* = 3 villages; rural with low exposure), northwest Upolu (*n* = 3 villages; periurban with moderate exposure), and apia urban area (*n* = 4 villages; urban area, high exposure). Primary caregivers and children were eligible to participate if both were of Samoan ethnicity based on primary caregiver-report of four Samoan grandparents and children were 2–4 years old at enrolment. in 2017–2018, we followed up with children from 2015 (*n* = 277; 3.5–8 years), surveyed an additional apia urban area village, and recruited additional eligible children to expand the cohort to 501 children in 11 villages. in 2019–2020, we completed follow-up assessments of 440 children aged 5 to 11 years (87.8% retention) whose data are reported here. Three children were excluded from the current analysis based on a lack of complete physical measurement data, resulting in a final analytic sample of 437 children.

### Data collection

Child characteristics were reported by primary caregivers. anthropometric measurements were collected using standardised procedures (US CDS 2013). measurements were administered in duplicate and averaged for analysis. Weight was measured to the nearest 0.1 kg using a Tanita HD 351 digital scale (Tanita corporation of america, IL), and height to the nearest 0.1 cm using a portable stadiometer (Pfister imports, NY). a finger-prick blood haemoglobin (Hb) sample was collected using an AimStrip test system (Germaine laboratories inc., TX).

### Measures

Age was calculated in years by subtracting the reported birth date from the date of the physical assessment. Sex assigned at birth was reported as either female or male. Dichotomous indicators of overweight/obesity, moderate/severe underweight, and moderate/severe stunting were created using the WHo child growth references based on sex and age at 5–11 years (BMI-for-age z-score > +1, weight-for-age z-score < −2, and height-for-age z-score < −2, respectively)(Onis et al. 2007). Degrees of severity were further categorised for underweight and stunting using z-score ranges of ≥ −2 to < −1(mild), ≥3 to < −2(moderate), and < −3 (severe) based on the WHO references (onis et al. 2007) and this has been used in other low-and-middle-income settings (Stevens et al. 2012). The WHO does not provide weight-for-age reference data beyond age 10 (WHO 2022). The presence or absence of any anaemia as a binary variable (Hb < 11.5 g/dl), and as an ordinal categorical variable was based on varying degrees from mild (11 < Hb < 11.5 g/dl) to severe (Hb < 8 g/dl) using the WHO cut-offs for children aged 5–11 years old (WHO 2011).

Data collection procedures were approved by yale and Brown university institutional review Boards (Hic# 2000020519, IAA# 18–41 959) and the Samoa ministry of Health, Health research committee. Written informed consent was obtained from primary caregivers and assent from children aged 7 years or older.

### Data management and statistical analysis

Prevalence of malnutrition (as binary and ordinal variables) was examined by child age (in years and 1-year age groups), sex, and census region using Wilcoxon two-sample tests for continuous variables, and chi-square tests or Fisher’s exact tests for categorical variables (when ≤20% of expected cell counts are <5, or >20% of expected cells counts are <5 respectively). Since no children were classified with severe degrees of underweight (WAZ< −3 SD) or stunting (HAZ < −3 SD), we presented on mild and/or moderate degrees of these outcomes to enhance the comparability of our findings with other studies in low-and-middle-income settings (Stevens et al. 2012). considering the prior literature (choy et al. 2017; choy et al. 2022), associations between malnutrition forms (overweight/obesity and anaemia) were assessed using chi-square tests. Statistical analyses were performed using SASversion 9.4 (SASinstitute inc., cary, nc). all reported probabilities (*p* values) were two-sided, with *p* < 0.05 considered meaningfully different (amrhein et al. 2019).

## Results

**Figure 1.**
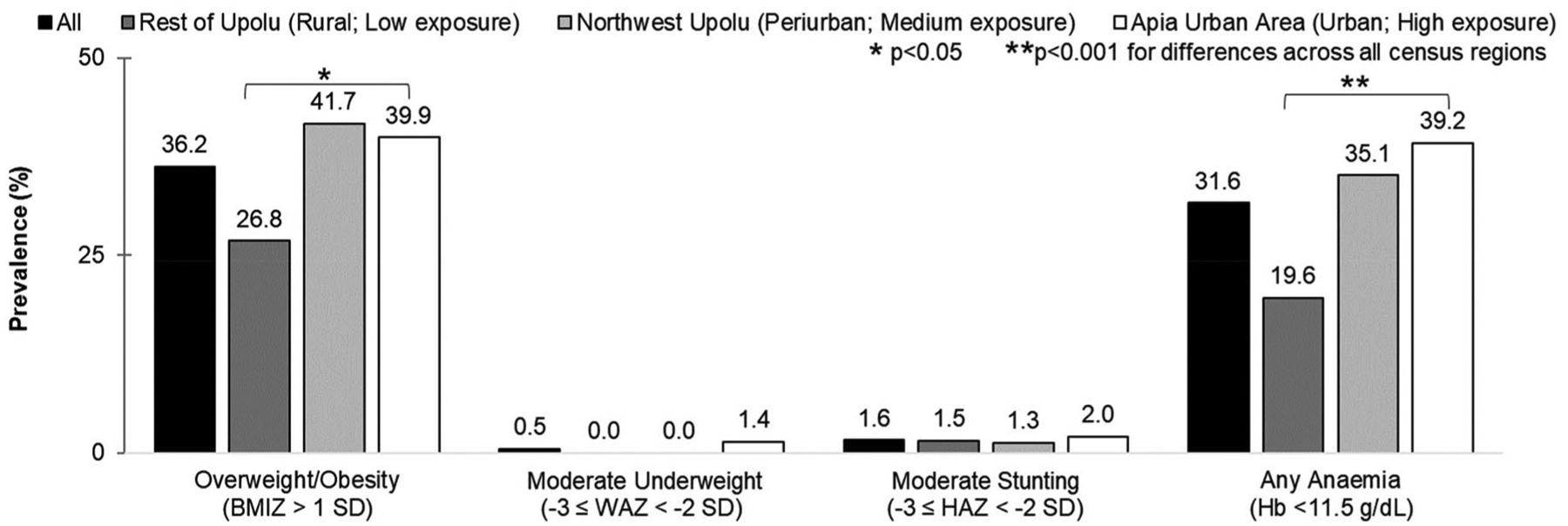
Prevalence of overweight/obesity, moderate underweight, moderate stunting, and any anaemia in children at age 5–11 years, by census region of residence. BMIZ: Body mass index-for-age Z-score (SD); WAZ: Weight-for-age Z-score (SD); HAZ: Height-for-age Z-score (SD); Hb: haemoglobin (g/dl). Chi-square tests or fisher’s exact tests were used to assess differences in prevalence by census region of residence. The census region categories represent varying urbanicity and exposure to nutrition transition (black: all children, dark grey: children living in the most rural region with low exposure in the Rest of Upolu; grey: children living in the periurban region with medium exposure in northwest Upolu; white: children living in the urban region with high exposure in Apia urban Area). overweight/obesity, underweight, and stunting were classified based on WHO child growth references Z-scores. no children were classified with severe degrees of underweight (WAZ< −3 SD) or stunting (HAZ < −3 SD).

The sample prevalence of overweight/obesity and any anaemia was 36.2% (*n* = 158) and 31.6% (*n* = 138), respectively (Figure 1). only seven children had height-for-age values indicative of stunting from mild to moderate degrees (1.6%) and two children were classified as underweight (0.5%; none severely underweight). approximately 10.3% (*n* = 45) of the children were affected by both overweight/obesity and anaemia, but no association was observed between these forms of malnutrition (*p* = 0.294). overweight/obesity and anaemia were positively associated with census region of residence, representing urbanicity and exposure to nutrition transition (*p* = 0.025 and *p* < 0.001, respectively). The lowest prevalence of overweight/obesity and anaemia was observed in the rural rest of ‘Upolu region with low exposure to the nutrition transition (26.8% and 19.6%, respectively).

Of the children with overweight/obesity, the majority (*n* = 84, 53%) were in the 8–9-year age groups (*p* = 0.012, Table 1). no sex differences were observed in any form of malnutrition prevalence. The prevalence of stunting of any degree from mild to moderate was higher among children living in the rural and periurban regions compared to the urban region (*p* = 0.036), while anaemia in any degree from mild to severe was highest in the periurban and urban regions (*p* = 0.008).

## Comment

This study identified more than one-third of the sample with some degree of overweight/obesity or anaemia at age 5–11 years in 2019–2020, highlighting the need for comprehensive childhood intervention strategies to reduce the population-level double burden in this setting. The prevalence of overweight/obesity in this sample at age 5–11 years was nearly double the prevalence reported in the same cohort at age 2–4 years (16.1%), while anaemia prevalence was similar (34.1%, choy et al. 2017). although anaemia is not necessarily persistent in the same children of this cohort (Wu et al. 2021), the WHO cautions that even mild levels of anaemia may impede adequate growth and development in children (WHO 2011), and in combination with obesity, may exacerbate the severity of nutrition-related non-communicable diseases (Tzioumis and adair 2014). The meaningful differences in the prevalence of overweight/obesity, stunting, and anaemia by census region suggest that at ages 5–11 years, children’s risk of malnutrition varies based on differential exposure to the nutrition transition and urbanisation. in prior research, children and adults living in the urban region had higher adherence to the modern dietary pattern compared to those in more rural regions (Wang et al. 2017; choy et al. 2020). This is in line with prior literature in other low-and-middle-income settings, where nutritional transitions towards increased sedentary activities and consumption of energy-dense, imported, and micronutrient-poor foods have been associated with both overweight/obesity and undernutrition (monteiro et al. 2004; Popkin et al. 2012). moreover, the findings highlight the continued role of environmental risk factors in the development of childhood obesity in this setting.

As with any study, there are limitations to consider in data interpretation. given the nature of this Human Biological Survey, we presented descriptive statistics documenting the prevalence of malnutrition in our sample. Future studies may look at the risk for malnutrition by various demographic, behavioural, and social characteristics to better understand what factors are associated. While there are children WHO experience both obesity and anaemia in the cohort (in which case, anaemia may be inflammation rather than nutrition-related), no prospective association between these outcomes has been observed in Samoan children (choy et al. 2022) and the causes of the double burden of malnutrition require further investigation. We used 2007 WHO growth references, which were developed based on child data collected in the united States (onis et al. 2007), to categorise children as having overweight/obesity, stunting, and underweight. These references may not account for potential differences in body composition and growth among populations in the Pacific compared to the WHO reference group (rush et al. 2008) so the later health impacts of these conditions may differ from those reported in the literature from other settings. a strength of this approach, however, is that WHO Z-scores are commonly used to inform public health efforts and allow for comparisons of our data with other populations around the world. as a result of the convenience sampling used to recruit the cohort and the cross-sectional nature of this present analysis, the prevalence estimates of malnutrition may not be generalisable to the population in Samoa and causality may not be inferred from their observed associations with the census region of village residence. although the cohort includes families with lower reported household assets compared to the general population (choy et al. 2017), the findings may be generalisable to children living in low socioeconomic resource households and WHO are among the most at risk of malnutrition in all forms. Further longitudinal investigation is necessary to understand the consequences of childhood malnutrition on health and well-being later in life and to inform public health policies and interventions that support health promotion, prevention, and healthcare access for children. an additional follow-up of children from this cohort is ongoing and future analyses will prioritise identifying modifiable predictors of overweight/obesity, anaemia, and related cardiometabolic diseases to improve public health intervention strategies.

## Figures and Tables

**Table 1.** Prevalence of malnutriton by child age, sex, and census region.

	Overweight/Obesity,* median (Q1-Q3) or *n* (%)				Underweight,* median (Q1-Q3) or *n* (%)				Stunting,* median (Q1-Q3) or *n* (%)				Anaemia,† median (Q1-Q3) or *n* (%)				
	None	Overweight	Obesity		None	Mild	Moderate		None	Mild	Moderate		None	Mild	Moderate	Severe	
	BMI *Z*≤ 1	2 ≥BMI *Z*>1	BMI *Z*> 2	Pǂ	WAZ ≥ −1	−1 > WAZ ≥ −2	−2>WAZ ≥ −3	Pǂ	HAZ ≥ −1 -	−1> HAZ ≥ −2	−2> HAZ ≥ −3	Pǂ	Hb ≥ 11.5	11.5> Hb ≥ 11	11> Hb ≥ 8	Hb < 8	Pǂ
Age (years)	7.9 (7.2–9.0)	8.0 (7.3–8.9)	8.5 (7.8–9.3)	0.012	8.0 (7.3–8.8)	7.7 (7.1–8.7)	8.6 (7.4–9.8)	0.386	8.0 (7.3–9.0)	7.9 (7.3–8.7)	9.1 (7.1–10.2)	0.458	8.1 (7.3–9.0)	7.8 (7.1–8.9)	8.0 (7.2–8.8)	7.6 (7.1–8.5)	0.379
5.0–5.9	7 (87.5)	0 (0.0)	1 (12.5)		8 (100.0)	0 (0.0)	0 (0.0)		8 (100.0)	0 (0.0)	0 (0.0)		5 (62.5)	2 (25.0)	1 (12.5)	0 (0.0)	
6.0–6.9	39 (76.5)	9 (17.7)	3 (5.9)		48 (94.1)	3 (5.9)	0 (0.0)		45 (88.2)	5 (9.8)	1 (2.0)		26 (51.0)	10 (19.6)	15 (29.4)	0 (0.0)	
7.0–7.9	104 (66.2)	37 (23.6)	16 (10.2)		146 (93.0)	10 (6.4)	1 (0.6)		140 (89.2)	16 (10.2)	1 (0.6)		113 (72.0)	19 (12.1)	23 (14.7)	2 (1.3)	
8.0–8.9	65 (56.5)	26 (22.6)	24 (20.9)		111 (96.5)	4 (3.5)	0 (0.0)		101 (87.8)	13 (11.3)	1 (0.9)		78 (67.8)	15 (13.0)	20 (17.4)	2 (1.7)	
9.0–9.9	48 (58.5)	16 (19.5)	18 (22.0)		79 (96.3)	2 (2.4)	1 (1.2)		76 (92.7)	4 (4.9)	2 (2.4)		58 (70.7)	9 (11.0)	15 (18.3)	0 (0.0)	
10.0–10.9	14 (70.0)	4 (20.0)	2 (10.0)		3 (100.0)	0 (0.0)	0 (0.0)		16 (80.0)	3 (15.0)	1 (5.0)		16 (80.0)	3 (15.0)	1 (5.0)	0 (0.0)	
11.0–11.9	2 (50.0)	1 (25.0)	1 (25.0)		-	-	-		3 (75.0)	0 (0.0)	1 (25.0)		3 (75.0)	0 (0.0)	1 (25.0)	0 (0.0)	
Sex				0.263				0.674				0.484					0.146
Female	142 (66.1)	48 (22.3)	25 (11.6)		197 (96.1)	7 (3.4)	1 (0.5)		193 (88.9)	19 (8.8)	5 (2.3)		147 (68.4)	33 (15.4)	35 (16.3)	0 (0.0)	
Male	137 (61.7)	47 (21.2)	38 (17.1)		198 (93.8)	12 (5.7)	1 (0.5)		198 (89.2)	22 (9.9)	2 (0.9)		152 (68.5)	25 (11.3)	41 (18.5)	4 (1.8)	
Census region				0.089				0.103				0.036					0.008
Rest of Upolu (Rural; Low exposure)	101 (73.2)	22 (15.9)	15 (10.9)		121 (95.3)	6 (4.7)	0 (0.0)		122 (88.4)	14 (10.1)	2 (1.5)		111 (80.4)	11 (8.0)	15 (10.9)	1 (0.7)	
Northwest Upolu (Periurban; Medium exposure)	89 (58.9)	36 (23.8)	26 (17.2)		134 (93.1)	10 (6.9)	0 (0.0)		128 (84.8)	21 (13.9)	2 (1.3)		98 (61.9)	20 (13.3)	31 (20.5)	2 (1.3)	
Apia Urban Area (Urban; High exposure)	89 (60.1)	37 (25.0)	22 (14.9)		140 (96.6)	3 (2.1)	2 (1.4)		139 (93.9)	6 (4.1)	3 (2.0)		90 (60.8)	27 (18.2)	30 (20.3)	1 (0.7)	

Abbreviations: BMIZ: body mass index-for-age Z-score (SD); WAZ: Weight-for-age Z-score (SD); HAZ: Height-for-age Z-score (SD); Hb: haemoglobin (g/dL).

* Based on the World Health organization (WHO) Child References (De Onis et al., 2007). Weight-for-age reference data are not available beyond age 10 years (WHO, 2022). No children were classified with severe degrees of underweight (WAZ< −3 SD)or stunting (HAZ < −3 SD)
